# Computer simulations of the signalling network in FLT3 ^+^-acute myeloid leukaemia – indications for an optimal dosage of inhibitors against FLT3 and CDK6

**DOI:** 10.1186/s12859-018-2145-y

**Published:** 2018-04-24

**Authors:** Antoine Buetti-Dinh, Ran Friedman

**Affiliations:** 1 Department of Chemistry and Biomedical Sciences, Linnæus University, Norra vägen 49, Kalmar, SE-391 82 Sweden; 2 Linnæus University Centre for Biomaterials Chemistry, Linnæus University, Norra vägen 49, Kalmar, SE-391 82 Sweden; 3 Centre for Ecology and Evolution in Microbial Model Systems, Linnæus University, Landgången 3, Kalmar, SE-391 82 Sweden; 4 Institute of Computational Science, Faculty of Informatics, Università della Svizzera Italiana, Via Giuseppe Buffi 13, Lugano, CH-6900 Switzerland; 5 Swiss Institute of Bioinformatics, Quartier Sorge – Batiment Genopode, Lausanne, CH-1015 Switzerland

**Keywords:** Acute myeloid leukaemia, Drug resistance, Knowledge-based analysis, Combination therapy

## Abstract

**Background:**

Mutations in the FMS-like tyrosine kinase 3 (FLT3) are associated with uncontrolled cellular functions that contribute to the development of acute myeloid leukaemia (AML). We performed computer simulations of the FLT3-dependent signalling network in order to study the pathways that are involved in AML development and resistance to targeted therapies.

**Results:**

Analysis of the simulations revealed the presence of alternative pathways through phosphoinositide 3 kinase (PI3K) and SH2-containing sequence proteins (SHC), that could overcome inhibition of FLT3. Inhibition of cyclin dependent kinase 6 (CDK6), a related molecular target, was also tested in the simulation but was not found to yield sufficient benefits alone.

**Conclusions:**

The PI3K pathway provided a basis for resistance to treatments. Alternative signalling pathways could not, however, restore cancer growth signals (proliferation and loss of apoptosis) to the same levels as prior to treatment, which may explain why FLT3 resistance mutations are the most common resistance mechanism. Finally, sensitivity analysis suggested the existence of optimal doses of FLT3 and CDK6 inhibitors in terms of efficacy and toxicity.

**Electronic supplementary material:**

The online version of this article (10.1186/s12859-018-2145-y) contains supplementary material, which is available to authorized users.

## Background

Predictive modelling approaches are used frequently during modern drug development. These include molecular modelling and screening [1], QSAR [2, 3], chemoinformatics-based ligand identification [4, 5], prediction of ADMET [6] and other aspects such as crystal structures of drugs [7]. Another important aspect is that of drug resistance, which is common in infectious diseases [8, 9] and cancer [10]. Unfortunately, our understanding of drug resistance and the causes for it is limited, and predictive approaches are hard to come by.

Many membrane-bound receptor tyrosine kinases (RTKs) are important for regulation of cellular growth [11, 12]. Mutations that alter their activity thus lead to abnormal proliferation that is associated with the development of cancers [13]. FLT3 is an RTK, whose physiological role is to regulate haematopoiesis. Mutations in FLT3 are involved in AML (FLT3 ^+^-AML) and, to a minor extent, in acute lymphoblastic leukaemia (ALL) as well [11]. This makes FLT3 a potential molecule drug target. Internal tandem duplications (ITD) in the juxtamembrane domain of FLT3 are common in FLT3-derived AML patients [14]. In addition, several mutations in the kinase activation domain cause sustained FLT3 activity that leads to uncontrolled proliferation and abates apoptosis. These include mutations in residues R834 [15], D835 [16], I836 [17], N841 [18] and Y842 [19] of the activation loop and rare mutations in the extracellular juxtamembrane domain [15]. Small molecules such as lestaurtinib, midostaurin, ponatinib, quizartinib, sorafenib, sunitinib and tandutinib can inhibit FLT3. Midostaurin has been recently approved by the US Food and Drug Administration (FDA) for the treatment of adult patients with newly diagnosed AML who are FLT3 mutation-positive. Ponatinib, sorafenib and sunitinib are approved for clinical use (for other conditions). Unfortunately, RTK inhibitors are often subject to drug resistance [20]. Known resistance mechanisms against midostaurin include FLT3-ITD overexpression, genetic (13q) alterations, upregulation of antiapoptotic genes, downregulation of proapoptotic genes, and FLT3 resistance mutations [21] including F621L, A627P, N676K, F691L, and Y842C [22, 23]. Alternative signalling can also provide cancers with treatment escape routes that bypass the signalling pathways blocked by therapeutic inhibitors. This resistance mechanisms is based on the fact that biological signalling is typically distributed over multiple components. It rarely relies on a single path that connects a receptor to its target, but rather involves multiple, converging, diverging and recursive branches of the signalling network. This provides means to cancers for boosting alternative signalling in order to compensate for pathways blocked by inhibitors, thereby promoting cancer-driving processes such as cellular proliferation or reduced apoptosis despite therapy [24, 25].

Experimental evidence indicates that FLT3 signalling induces a cascade of events that involves an intricate network of signalling components comprising CDK6, PI3K, STAT (signal transducer and activators of transcription), AKT (protein kinase B), BCL2-BAD (BCL2-family protein – BCL2 antagonist of cell death), RAS, MEK/ERK (mitogen-activated ERK kinase / extracellular signal-regulated kinase) and other cellular components known to play a role in the development of diverse cancers [11, 12, 14, 26–37]. Following how individual components of the signalling networks interact in a cancer cell is a challenge. We have developed a computational framework to study signal transduction networks based on chemical principles [38]. Through interfering with some of the network components, we identified conditions in which interventions to prevent metastasis in a model breast cancer could work (or not) [39], and suggested combination therapy for nucleophosmin anaplastic lymphoma kinase (NPM-ALK) derived anaplastic large cell lymphomas [40]. Other approaches exist to analyse signal transduction networks with different degrees of details necessary to set up a model [41, 42] from highly detailed (e.g., based on mass-action kinetics) [43–48] to qualitative Boolean models [49, 50]. In between these two extremes, semi-quantitative models make simplifying assumptions that allow to provide quantitative insights on the studied system, while requiring fewer experimental details to set them up [38–40, 51, 52]. The epidermal growth factor receptor ErbB signalling network was analysed by integrating high-level details into a mass-action-based modelling framework and therapeutic antibodies to target the cancer-related ErbB3 RTK were developed [45, 46, 48]. New combination therapies were also suggested by semi-quantitative models of AML signalling [51]. An advantage of semi-quantitative models is that their flexibility allows to take into account aspects of cellular communication networks that are increasingly recognised to play a role in cancer development and emergence of resistance to therapies. This allows to perform simulations of cell signalling that include the evolution of cancer cell populations [20, 53–55], cellular heterogeneity [56–60], and the selective pressure in the cancer microenvironment [61].

Since midostaurin has only been approved for clinical use this year and given that FLT3 ^+^-AML is a fairly rare cancer, little is known on alternative signalling pathways or the potential for combination therapy. We applied a knowledge-based numerical simulation and sensitivity analysis to different FLT3 network models. Our aim was to assess the effect of single or dual therapeutic inhibition. This allowed us to make predictions on signalling pathways that are liable to confer resistance to therapy aimed at FLT3 ^+^-AML. The networks were analysed with respect to apoptosis and cell proliferation, where loss of apoptosis (LOA) and gain of proliferation were viewed as cancer promoting end-states. Interestingly, it has been suggested before that apoptosis can be important for cancer progression if cell division is slow [62]. In the case of AML, however, this does not appear to be the case, i.e., inhibition of apoptosis promotes survival of the cancer cells [63].

## Results and discussion

**Fig. 1:**
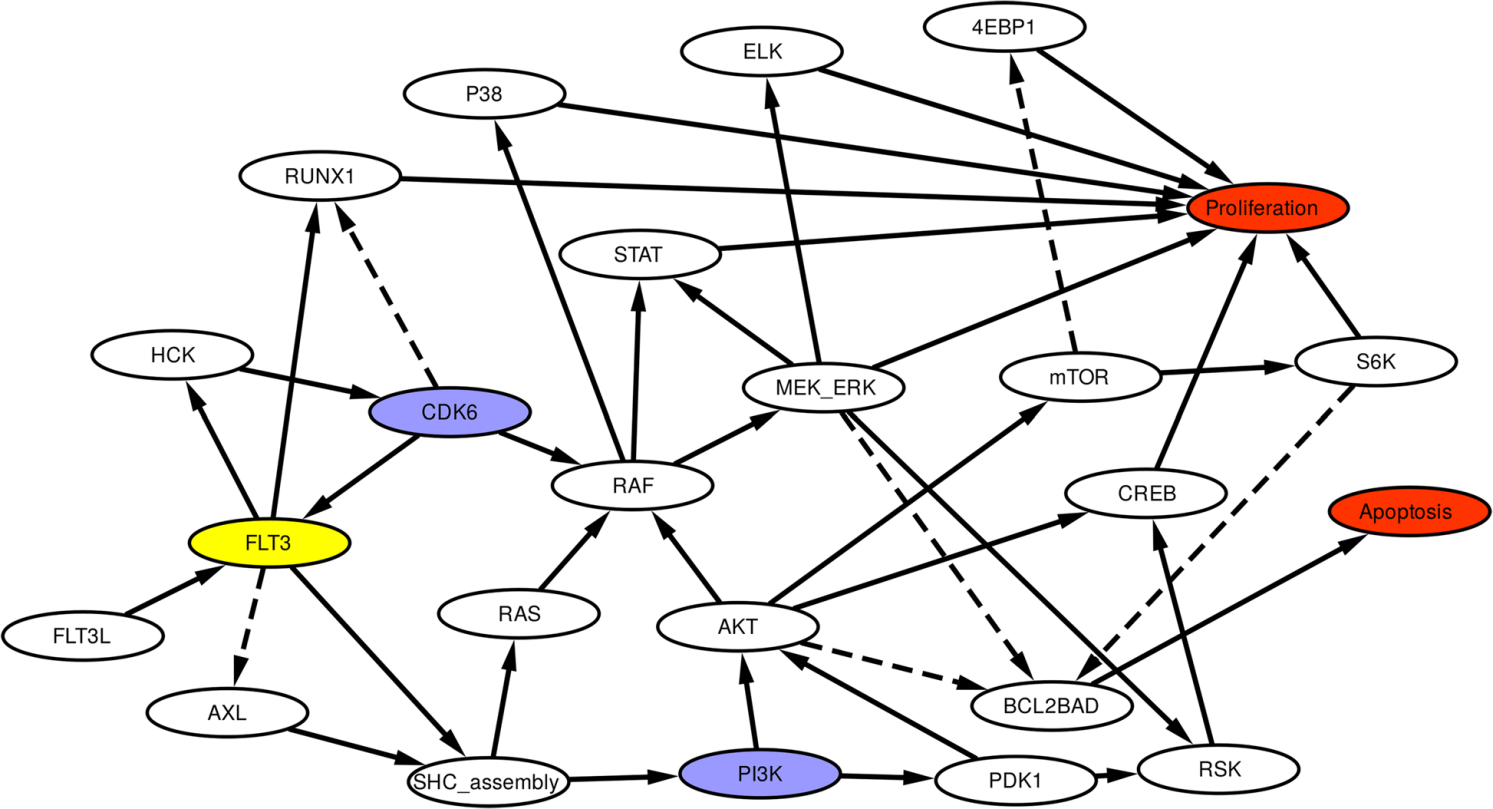
The interaction network of FLT3. FLT3 is represented in yellow and through different nodes it transduces the signal to *proliferation* and *apoptosis*, the network’s end-points that contribute to the development of AML (red nodes). Blue nodes represent potential candidates for combined inhibition therapy. Note that the two end-points yield different consequences: proliferation leads to tumour growth, whereas apoptosis limits the growth (and thus LOA leads to tumour growth)

The network of interactions in FLT3 ^+^-AML is presented in Fig. 1. The signals are transmitted between the different components of the network through activation or inhibition, which results in two cancer-promoting end-states: increased cell proliferation and LOA. The simulations were first performed by applying a coarse-grained approach [40] whereby each node assumed one of two possible states (“low activity” or “high activity”), and exhaustive simulations were performed (see the “Methods” section).

The approach was applied to four FLT3 network variants: an intact (complete) network (“FLT3, FLT3-ligand, CDK6 and HCK contribute the most to cell proliferation and loss of apoptosis” section), a network with constitutive low activity of FLT3 (simulating FLT3 targeted inhibition, “Inhibition of FLT3 intensifies signal flow through SHC, PI3K, RAS, AKT and PDK1” section), network with constitutive low activity of CDK6 (simulating CDK6 targeted inhibition, “FLT3, SHC and PI3K are important for the control of end-points when CDK6 is inhibited” section), and constitutive low activity of FLT3 and CDK6 (dual inhibition, “Combined inhibition of FLT3 and CDK6 may be overcome through SHC and PI3K signalling” section). Sensitivity profiles of each network (central plots in Additional file 1: Figures S1–S4) were obtained by simulating all combinations of network states where each node’s activity could be either high or low. These sensitivity plots represent how sensitive the end-points (proliferation or apoptosis) are to modification of the activity of each of the other nodes, which suggests potential modes of intervention. A subset of network states, corresponding to the upper and lower extremes of sensitivity profiles, represents network components that strongly contribute to change the cancer-promoting end-states (increased cellular proliferation and LOA, represented by the red and blue datapoints in the central plots of Additional file 1: Figures S1–S4, respectively). Bar plots flanking the central sensitivity plot represent the relative percentage of cases where a node was responsible for a high or low sensitivity value among all network states constituting the top/bottom-2%. In addition, the most probable signalling path from the most influential nodes to the end-points was also inferred (signal flow graphs on the top and bottom of Additional file 1: Figures S1–S4). The coarse-grained analysis was later complemented by detailed (fine-grained) simulations where few nodes assumed multiple intermediate activity values while the others assumed low (resting) activities.

### FLT3, FLT3-ligand, CDK6 and HCK contribute the most to cell proliferation and loss of apoptosis

A first set of simulations was performed with the intact network in order to identify the components that contribute the most to increased cell proliferation and LOA. This analysis (Additional file 1: Figure S1) revealed that FLT3, FLT3-ligand (FLT3L), HCK (hematopoietic cell kinase) and CDK6 were those nodes that were most commonly associated with both end-points. FLT3L is a hematopoietic growth factor that activates wild-type (wt)-FLT3 [64]. Constitutively active FLT3 (due to driver mutations or ITD) does not depend on FLT3L. This is clearly shown in the simulations when examining signal transduction under the conditions during which FLT3 was the cause of an increased cell proliferation or LOA (signal flow graphs on the top- and bottom left-hand sides of Additional file 1: Figure S1). In these graphs, the statistical association of other nodes involved in the end process simultaneously with FLT3 is indicated by the graph’s node sizes (the larger the stronger the association). The colour of the nodes indicates their activity contribution (the darker is the node, the stronger is its ability to deliver a signal downstream to it). As shown in these graphs, when FLT3 is highly active, HCK, CDK6 and RUNX1 (runt-related transcription factor 1) are also highly active, but FLT3L is not. The nodes that play a major role in developing a proliferative phenotype when FLT3 is turned on include HCK, CDK6, SHC, RUNX1 (top graphs in Additional file 1: Figure S1). A similar situation was observed for the graphs associated to LOA. However, as indicated by the bottom-2% bar plot, with the difference that PI3K together with its downstream nodes (AKT, PDK1, RSK (90-kDa ribosomal protein S6 kinase), CREB (cyclic adenosine monophosphate-response element binding protein), and mTOR (mammalian target of rapamycin)) can become an alternative pathway to LOA (bottom right graphs in Additional file 1: Figure S1).

Our simulations agree with experimental data obtained using AML cell lines carrying FLT3-ITD mutations which were subject to small interfering RNA (siRNA) inhibiting FLT3 or HCK. This caused a reduction in proliferation of ∼ 3–10-fold [14]. Similarly, in our coarse-grained simulations, when inhibiting *in silico* FLT3 we could observe a decrease in frequency of CDK6 and HCK of ∼ 10-fold in the top-2% regions of the proliferation sensitivity profile (Additional file 1: Figures S1–S2).

HCK is a non-RTK which is highly expressed and activated in some leukaemias but whose expression is reduced in others [65]. HCK can be inhibited by small molecules such as RK-20449 [66], which may have beneficial effects against several cancers [66, 67]. CDK6 is a serine/threonine protein kinase that contributes to the entry of the cell to the DNA synthesis phase (G1 →S) of the cell cycle. The CDK6 inhibitors palbociclib and ribociclib are used in the treatment of advanced-stage oestrogen receptor (ER)-positive breast cancer [68] and may be used in other cancers as well (including AML [69]). Resistance mutations to palbociclib have hitherto not been detected, perhaps due to its binding mode [70]. Thus, both CDK6 and HCK may be relevant drug targets in FLT3 ^+^-AML in addition to FLT3. CDK6 inhibitors have the advantage that they are already approved and considered safe to use.

### Inhibition of FLT3 intensifies signal flow through SHC, PI3K, RAS, AKT and PDK1

Following the simulation of the intact signalling network, a second set of coarse-grained simulations was performed, this time by inhibiting FLT3. The results of these simulations are presented in Additional file 1: Figure S2. The bar plots in the figure indicate that, upon inhibition of FLT3, the most important signal transduction components become the adapter protein Shc (SHC), the cell surface RTK AXL, and PI3K. AXL was found to be more relevant to proliferation in this case, and PI3K to LOA. Interestingly, inhibition of FLT3 removes the influence of HCK and CDK6 on the end-points. This is likely due to the feedback loop involving CDK6, FLT3 and HCK.

Simulations of the network were also used to follow on the signal flow. This analysis revealed that inhibition of FLT3 resulted in an intensification of the flow through SHC, PI3K, RAS, AKT and PDK1. Apparently, PDK1 and AKT could activate an alternative signalling pathway to stimulate proliferation (top 4^th^ and 5^th^ graphs in Additional file 1: Figure S2). This corroboration from the simulations is supported by qualitative experimental data within the development of BAG956 inhibitor [71, 72] However, the influence of these nodes was rather limited, as indicated by the corresponding bar plot (frequency < 10%). This could explain why the most common resistance mechanism to FLT3 inhibitors is resistance mutations. Apparently, alternative networks only partially restore the signal to proliferation and LOA.

### FLT3, SHC and PI3K are important for the control of end-points when CDK6 is inhibited

Since CDK6 inhibitors are available, tolerated and hitherto not subject to resistance mutations, inhibition of CDK6 was also simulated as an alternative to inhibition of FLT3 (Additional file 1: Figure S3). Whereas inhibition of FLT3 reduced the significance of CDK6, CDK6 inhibition did not have the same influence on FLT3, which remained a key component of the network in promoting proliferation, together with its ligand, SHC, AXL and PI3K. FLT3 is represented in 22% of the simulations where proliferation was highest, and only in those cases HCK was also important (signal flow graphs, top left). Otherwise, the feedback loop involving CDK6, FLT3 and HCK, is inactive and signalling is compensated by the nodes in the lower part of the graphs (FLT3, AXL, SHC, RAS and PI3K). The involvement of these nodes compensates for the inhibition of CDK6 and suggests that proliferation can be stimulated through PI3K, SHC and AXL in alternative to the intact network signalling. Experimental data support our simulations except for the role of the SRC kinase (included in the SHC_assembly node of our model) shown to also influence CDK6, not acting only downstream of it [14]. This is possibly due to the promiscuous nature by which SH domains bind their partners to assemble diverse molecular complexes [73].

With respect to LOA, when CDK6 was inhibited, the role of FLT3 became much less important. Instead, PI3K took over. Taken together, the simulations with inhibited CDK6 indicated that PI3K, SHC and AXL became signalling alternatives for both proliferation and apoptosis. Interestingly, PI3K was suggested to be an escape mechanism for ER positive breast cancer tumours that became resistant to CDK6 inhibitors [14, 74]. This may be a common escape mechanism for CDK4/6 inhibitors.

### Combined inhibition of FLT3 and CDK6 may be overcome through SHC and PI3K signalling

The simulations of FLT3 inhibited and CDK6 inhibited networks indicated that FLT3 inhibition had a larger effect than CDK6 inhibition, and that FLT3 was important for proliferation even if CDK6 was inhibited. In a final set of coarse-grained simulations, both FLT3 and CDK6 were inhibited (Additional file 1: Figure S4). By and large, the results of the simulations with dual inhibition of FLT3 and CDK6 resembled the case of FLT3 inhibition, where stimulation of proliferation and apoptosis were dependent almost exclusively on SHC and PI3K signalling. A notable difference, however, was the emergence of MEK/ERK in the proliferation bar plot (albeit at a low influence level (< 5%), see Additional file 1: Figure S4).

### Sensitivity analysis suggests that the system can be controlled even if PI3K expression is increased

**Fig. 2:**
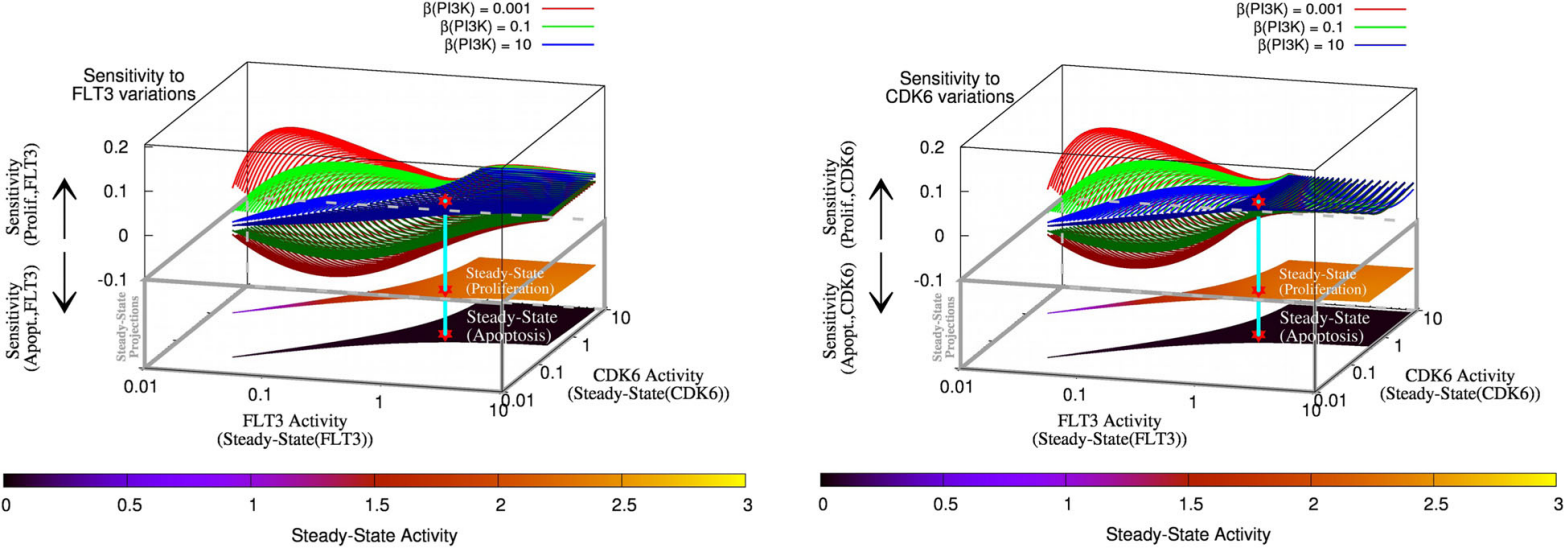
Fine-grained simulations. Steady-state and sensitivity of proliferation and apoptosis to variations of the activities of FLT3 and CDK6. Convex (concave) surfaces represent the sensitivity of the proliferation (apoptosis) end-point with respect to variation in FLT3 (left) or CDK6 (right) activities. The bottom projections in the lower planes (within the gray box) are set to arbitrary z-axis values and represent steady-state activities of the end-points as a function of FLT3 and CDK6 activities at low PI3K activity. These projections correspond to the sensitivity surfaces in the upper part and allow to visualise how the variables FLT3 and CDK6 depend on each other, and their influence on the network end-points (further details of such projections at different levels of PI3K activity are available in Additional file 1: Figure S5–S6). The red star symbols indicate the point where sufficient inhibition of FLT3 (10-fold inhibition from the maximum) and CDK6 (15-fold inhibition from the maximum) drive the system to a controllable region of intermediate steady-state levels of both proliferation and apoptosis. A cyan segment connects this point through the different complementary quantities represented, i.e., the sensitivities at different PI3K activities in the upper surfaces, and the corresponding end-points’ steady-state activities in the lower projections. This multidimensional representation allows to appreciate both the steady-state activity of the variables (which would correspond to experimentally measurable quantities such as tumour markers, RNA or proteins), as well as their sensitivity to changes in the other variables’ activities. The left and right plots can be compared to top-view heat maps for proliferation (Additional file 1: Figure S5) and apoptosis (Additional file 1: Figure S6) which represent steady-state and sensitivity. PCA of the network variables under different PI3K independent activities is shown as a function of PI3K activities in Additional file 1: Figures S7–S9

Fine-grained simulations are computationally demanding, but enable the calculation of sensitivity of the system with respect to small variations of the variables and identify regions that can be controlled through intervention (here, inhibition of FLT3, CDK6, or both) or where inhibition is not beneficial in terms of achieving the desired results. To this end, following earlier studies [38, 39], simulations of the networks were carried out where the activities of FLT3 and CDK6 were modified in small steps (see the “Methods” section) subject to three levels of PI3K activity i.e., normal, low (1/100 of the normal level), and high (100 × normal). The results of this analysis are shown in Fig. 2).

Analysis of the fine-grained simulations revealed that under the right conditions, the system could remain under control with respect to apoptosis and proliferation. A controllable region (sensitivity higher or lower than zero) was observed in the low to medium range of FLT3 and CDK6 activities (as shown by the sensitivity surfaces in Fig. 2, and Additional file 1: Figures S5–S6). Beyond that threshold (i.e., where sensitivity is close to zero), the system lost controllability to external stimuli, and a high proliferation regime became dominant (as presented by the upper x-y-plane projections in plots of Fig. 2). Loss of controllability of LOA was observed at the same time, but to a smaller extent (as shown by the lower x-y-plane projections in Fig. 2 and more clearly in Additional file 1: Figure S6). Increasing the activity of PI3K decreased the end-points’ sensitivity to changes in the activities of FLT3 and CDK6. This made the system less controllable by external stimuli (Fig. 2). Moreover, once high proliferation and LOA are reached, the simulations predicted that reverting back to a physiological, healthy regime will be difficult if at all possible through inhibition of FLT3 and CDK6.

### Unexpected connections between the nodes revealed by principal component analysis

Principal component analysis (PCA) was used to detect co-activity (when it was applied to steady-state values) and co-regulation (when it was applied to sensitivities) patterns between the signalling components in fine-grained simulations under different PI3K activities as above. The results of this analysis are presented in Additional file 1: Figures S7–S9 which correspond to the red, green, and blue curves, respectively, in Fig. 2.

In the steady-state PCA, FLT3 and CDK6 were clustered together because of the external *β* tuning (see Additional file 1: Table S1). RAF, SHC and HCK were clustered with the proliferation end-point at low and intermediate PI3K activity, while at high PI3K activity proliferation merged with a neighbouring cluster composed of STAT, PI3K and RAS. This suggests that proliferation becomes driven by STAT and RAS upon an increase in PI3K activity. The apoptosis end-point clustered with AXL, FLT3L, 4E-BP1 (eukaryotic initiation factor 4E-binding protein) and BCL2-BAD at low PI3K activity. It merged with other network components into larger clusters as the activity of PI3K was increased. This suggests that control of apoptosis with increased PI3K activity becomes distributed over multiple nodes besides the ones strictly belonging to the apoptosis signalling path (S6K and BCL2-BAD).

The sensitivity PCA indicated that FLT3 clustered with RUNX1 at all levels of PI3K, while CDK6 clustered with AXL at intermediate and high PI3K activity. Together, they were associated with apoptosis among other components (BCL2-BAD, 4E-BP1 and FLT3L at low PI3K). Interestingly, a cluster composed of RAS, SHC and HCK became isolated from the other variables at intermediate and high PI3K activity (increasing hierarchical clustering height) whereas the same components clustered with PDK1, PI3K and AKT at low activity of PI3K. This suggests that with increasing activity of PI3K, co-regulatory patterns become more defined in grouping FLT3 with RUNX1, CDK6 with AXL, PI3K with PDK1, and AKT, RAS with SHC and HCK. In contrast, the end-points proliferation and apoptosis clustered in small groups under low PI3K but merged into larger ones under higher activity levels of PI3K. This suggests that regulation of the end nodes at high activity of PI3K became distributed over a larger number of signalling components, which explains the loss of sensitivity observed in the sensitivity profiles as a function of increasing PI3K (Fig. 2).

### Combined inhibition of FLT3 and CDK6 can be beneficial

The feedback between FLT3 and CDK6 (Fig. 1) implies an interdependent regulation between FLT3 and CDK6, which has the effect of restricting the activity of these two components of the network to a similar range (as indicated by the diagonal narrow steady-state activity projections in the x-y-plane of Fig. 2 that expand in correspondence to high activity levels of FLT3 and CDK6). This pattern suggests that a combined, partial inhibition of FLT3 and CDK6 would be sufficient to restrict the system to a sensitive area of the regulation space represented in Fig. 2. More precisely, a 10-fold inhibition of FLT3 from its maximum activity level, combined with a 15-fold CDK6 inhibition from maximal CDK6 activity, would suffice to drive the system to a sensitive region of intermediate steady-state levels of both proliferation and apoptosis. This point (indicated by a red star symbol in Fig. 2, and Additional file 1: Figures S5–S6) corresponds to the transition zone between the region where sensitivity surfaces are close to zero, and therefore the system is poorly controllable, and the region of higher controllability where sensitivity surfaces have positive or negative values. Stronger inhibition of either or both components, is predicted to further decrease the activities of the cancer-driving end-points (in a synergistic way, due to the feedback loop involving FLT3, CDK6 and HCK). Combination of FLT3 and CDK6 inhibitors in smaller doses than required for individual therapy may thus be sufficient or even superior solution in terms of efficacy and minimisation of secondary effects.

## Conclusions

Simulations of the network of interactions based on the current knowledge of FLT3 ^+^-AML were carried out in order to identify potential routes of resistance besides FLT3 mutations and examine the potential for combined inhibition of FLT3 and CDK6. Although both FLT3 and CDK6 inhibitors are available, resistance and intolerance limit their benefits. Particularly, CDK6 inhibitors may not be tolerated due to toxicities [75]. FLT3 inhibitors have limited use due to the emergence of mutations that make the drugs less efficient in controlling the activity of FLT3.

The simulations suggested that upon FLT3 inhibition, signal flow through SHC, PI3K, RAS, AKT and PDK1 becomes more intense and may provide alternative paths to maintain sustained cellular proliferation and reduced apoptosis. Inhibition of CDK6 was of little use in itself since FLT3 could still drive cell proliferation. Combined inhibition of FLT3 and CDK6 reduced the severeness of cancer-promoting processes, but could still be bypassed by PI3K-mediated signalling involving the nodes PI3K, SHC and AXL resulting in potential treatment escape routes. The simulations indicated that FLT3, SHC and PI3K are important for the end-points’ control when CDK6 is inhibited. The analysis further suggests the existence of an optimal combination of FLT3 and CDK6 inhibitors that would be efficient even if FLT3 is somewhat more active due to resistance mutations and may require lower doses of CDK6 than necessary for inhibition of CDK6 alone.

## Methods

### FLT3 signalling network

A knowledge-based network model of FLT3 and its principal interaction partners was assembled by combining the experimental information summarised in references [11, 12, 14, 26–37]. FLT3 was shown to associate in vitro with the SHC complex (composed of SHC, CBL (a proto-oncogene), SHIP (SH2-domain-containing inositol phosphatase), SHP2 (SH2-domain-containing protein tyrosine phosphatase 2), GAB2 (GRB2-binding protein) and GRB2-SOS (son of sevenless) and lumped together in a single network node denoted as “SHC_assembly” in the network scheme (see Fig. 1), or as “SHC” (in Additional file 1: Figures S7–S9) [26–30]. Downstream of the SHC complex, the RAS → RAF → MEK/ERK pathway influences the activity of genes involved in stimulating cellular proliferation and repressing apoptosis. These cancer-driving processes are lumped together in two separate network end-points and are denoted in the network scheme as “*proliferation*” and “*apoptosis*”. ETS domain-containing protein (ELK), p38 and STAT mediate signalling from RAF/MEK/ERK to the transcription of genes involved in proliferation [11, 31] together with the PI3K → AKT pathway which regulates apoptosis as well through mTOR, S6K and BCL2-BAD [12, 32, 33]. The same pathway also bridges proliferation with apoptosis via PDK1, RSK and CREB [11, 12, 34]. Finally, interactions were included to take into account the regulation between FLT3, HCK and CDK6 [14, 35], as well as the role of RUNX1 and AXL kinases [36, 37].

### Network simulation and sensitivity analysis

Signalling in the FLT3 networks (intact network, inhibited FLT3, inhibited CDK6, inhibited FLT3 and CDK6) was simulated with the computational method developed by us previously [38, 39]. Signalling networks were constructed as interaction diagrams composed of nodes and edges. The nodes represented signalling components as a set of ordinary differential equations (ODEs). Edges represented the interaction links between the components (modelled as empirical Hill-type transfer functions). The system is described as a network of interacting components that evolve in time according to the ODEs. Every node in the model is parametrised by the parameters *β* and *δ* and every link by *α*,*γ* and *η* (see Table 1), resulting in a set of ODEs for the nodes {*X,Y*,...}:

1 $$ \left\{\begin{array}{ll} dX/dt = - \delta_{X}X + (\beta_{X} + \sum_{i} {Act}_{i}) \cdot \Pi_{j} {Inh}_{j} \\ dY/dt = - \delta_{Y}Y + (\beta_{Y} + \sum_{i} {Act}_{i}) \cdot \Pi_{j} {Inh}_{j} \\ \cdots \\ \end{array}\right.  $$

**Table 1:** Model parameters

Parameter Name	Description
*β*	Basal level of a node’s activity
*δ*	Decay constant of a node
*γ*	Interaction strength between two nodes
	(affinity)
*η*	Nonlinearity in signalling interaction
	(Hill coefficient)
*α*	Multiplicative scaling factor applied to the
	regulatory function

Parameters used to define model’s nodes and links

The parameter *β* accounts for the basal activity as a zero-order term added to each ODE, and *δ* for the decay of the biological species as a first-order decay term subtracted from the ODEs. We refer to the activity of a protein in analogy to the activity of a chemical solute, i.e., it corresponds to the effective concentration of a protein in its biologically active conformation. The biological activity cannot be compared directly with experiments and is given in arbitrary units that can be roughly translated to a signalling protein that is abundant in the cell (i.e., in the order of 1 *μ*M) [76]. Values for end-points (proliferation and apopotosis) can only be appreciated by comparison, and we assume that any treatment would aspire to keep proliferation as low as possible and apopotosis as high as in healthy physiological conditions.

The Hill-type regulatory functions used to link the nodes to each other are defined according to Eqs. 2 and 3 for activation and inhibition, respectively. Arrows representing activation (→) and inhibition ($\dashrightarrow $) correspond to the network scheme in Fig. 1.

2 $$\begin{array}{@{}rcl@{}} Act(X \longrightarrow Y;\alpha,\gamma,\eta) = \alpha\frac{X^{\eta}}{X^{\eta}+\gamma^{\eta}} \end{array} $$

3 $$\begin{array}{@{}rcl@{}} Inh(X \dashrightarrow Y;\alpha,\gamma,\eta) = \alpha\frac{\gamma^{\eta}}{X^{\eta}+\gamma^{\eta}} \end{array} $$

The Hill-exponent *η* is an empirical parameter widely used to quantify nonlinear signalling interaction (e.g., positive/negative binding cooperativity) [77] and was kept equal to one in the present work. The parameter *γ* establishes a threshold of activation along the abscissa and *α* is a multiplicative scaling factor and have been set to one throughout the current work. When multiple links point to a single node, activation functions are added to each other while inhibition functions are multiplied by the current level of activity (see references [78, 79]).

This modelling framework enabled the integration of experimental information in a straightforward way using a well-established formalism derived from classical enzyme kinetics and test different model variations, such as the (combined) inhibition of FLT3/CDK6 in the model. This approach requires only the knowledge necessary to set up Boolean models (where interaction is assumed to be binary, i.e., activation or inhibition). Yet it provides quantitative insights on the studied signalling networks, taking into account nonlinear signalling effects such as feedbacks, pleiotropy and redundancy.

The simulation procedure yielded steady-state activity levels of the different network components according to a given set of parameters. The steady-state of the ODEs system was calculated numerically using the GSL library [80] (by use of *gsl_odeiv2_step_rk4*, which employs the explicit 4^*th*^ order Runge-Kutta algorithm). With this procedure the steady-state values of each node is obtained for a given parameter set. The range of independent activities of the different network components (*β*) used, is summarised in Additional file 1: Table S1. Sensitivity analysis was applied to the resulting steady-state activities by calculating the sensitivity corresponding to each parameter change in the combinatorial parameter space according to

4 $$ {{\varepsilon}}^{Y}_{\phi} = \frac{\partial [ln(Y)]}{\partial [ln(\phi)]} = \frac{\phi}{Y} \cdot \frac{\partial Y}{\partial \phi} \approx \frac{\Delta [ln(Y)]}{\Delta [ln(\phi)]} = \frac{ln(Y_{i} / Y_{j})}{ln(\phi_{i} / \phi_{j})}  $$

where the sensitivity ${{\varepsilon }}^{Y}_{\phi }$ is represented as a function of the input parameter set *ϕ* and of the output variable *Y*. Equation 4 expresses the relative change of activity in the nodes as a function of varying parameter sets. Two conditions (*i* and *j*) are evaluated at each step of the computational procedure according to the right-hand approximation. Here, the conditions are represented by vectors of steady-state values (*Y*_*i*_ and *Y*_*j*_) that correspond to the nodes’ activities and are determined by the parameter sets (*ϕ*_*i*_ and *ϕ*_*j*_).

In order to reveal co-activity and co-regulatory patterns between the nodes in the multi-dimensional simulation data, the resulting steady-state activity and sensitivity values were further explored through multivariate analysis (see “Principal component analysis and hierarchical clustering” section).

Steady-state simulations and sensitivity analysis were carried out using parallel computational architectures in order to screen a large number of conditions and identify key control points of the different networks. This enabled us to methodically characterise the effect of inhibition of FLT3 and/or CDK6 in the different network models.

### Sensitivity analysis in coarse-grained simulations

Coarse-grained simulations consisted of enumerating all combinations of network states with *high* (*β*=0.1) or *low* (*β*=0.001) initial activity state (see Additional file 1: Table S1). Each pair of combinations that differed by a single parameter (i.e., where the network state differed by the activity of a single node), was used to compute the sensitivity (Eq. 4) of the given modification according to the method used in reference [40], i.e.,

5 $$ {}{{\varepsilon}}^{SS(N_{i})_{\beta(N_{j})=low} \: \rightarrow \: SS(N_{i})_{\beta(N_{j})=high} }_{{ \beta(N_{j})=low} \: \rightarrow \: \beta(N_{j})=high } = \frac{ ln \bigg \{ \frac{SS(N_{i})_{\beta(N_{j})=high} }{ SS(N_{i})_{\beta(N_{j})=low}} \bigg \} }{ ln \bigg \{ \frac{{\beta(N_{j})=high} }{{\beta(N_{j})=low}} \bigg \} }   $$

where *SS*(*N*) denotes the steady-state activity of a node *N* and *β*(*N*) its independent activity state. The arrow (→) indicates a change in condition.

Without considering the combined activity change of multiple control nodes simultaneously, but only the changes occurring subsequently one after another (as it would be expected by point mutations affecting the activity of a protein), Eq. 5 allows to calculate the *s*^*n*^ conditions that represent all possible states of the network (*s* is the number of states a node can assume, *n* is the number of nodes in the network).

Sensitivity is subsequently computed for each pair of simulated conditions that differ by a single parameter (i.e., pair of simulations where the network states are identical except for a single node that is low in the first simulation and high in the second, or *vice versa*). This resulted in a set of calculated sensitivities derived from the coarse-grained simulations that comprises $s^{n} \cdot \frac {n}{s} \cdot (s-1)$ sensitivity values from which sensitivity profiles and signal flow graphs are computed (see “Sensitivity profiles and signal flow graphs” section).

Each sensitivity value expressed the strength of a link between two components of the network, regardless of the degree of connection (directly or through intermediates). A positive value for the sensitivity between two nodes (A → B) indicated that upon the increase of the activity of A, B’s activity would also increase. Similarly, a negative sensitivity indicates that upon an increase of A’s activity, B’s activity would decrease. Sensitivity values close to 0 indicates independence between nodes.

#### Sensitivity profiles and signal flow graphs

We tested each possible combination of the network nodes (high or low initial activity), for each network simulated (intact, inhibited FLT3, inhibited CDK6, inhibited FLT3 and CDK6). The results are presented by “*sensitivity profile plots*” and “*signal flow graphs*”, as described below.

##### Sensitivity profile.

The central sensitivity profile plots in Additional file 1: Figures S1–S4 represent the sensitivity calculated for each network simulated by coarse-grained simulations, ranked in ascending order. The majority of the combinations had no effect on the network end-points. These are represented by the flat part of the plots (black for the end-point proliferation, grey for the end-point apoptosis). A minority of the sensitivity values were far from zero: the red dots represented positive values for proliferation, whereas the blue dots represented negative values for apoptosis (in this case, we consider that the cancer-driving process is LOA, therefore we observe a negative sensitivity). These values far from zero represent a subset of nodes which, upon their increased activity, significantly contribute to activate proliferation or inhibit apoptosis.

This subset of nodes responsible for high and low sensitivities (top-2% (red) and bottom-2% (blue) portion for proliferation and apoptosis, respectively) were further analysed to identify how strongly certain nodes were associated to proliferation and apoptosis. The bar plots connected to top-/bottom-2% regions of the sensitivity profile show the frequency of the nodes, that upon switching from low to high activity, contribute to increase proliferation (red) or decrease apoptosis (blue).

##### Signal flow.

Signal flow graphs connected to the bars of the bar plots represent how the signal travels from the control node (node indicated on the bar) to the end-points (top and bottom graphs in Additional file 1: Figures S1–S4) according to the method described in reference [40]. Briefly, we define “*control nodes*” as the nodes that, upon a change in their activity (owing to external or internal perturbations), would cause changes in the activity of the other nodes in the network. While end-point nodes contribute to the development of AML (red nodes in Fig. 1*proliferation* and *apoptosis*).

In order to examine pathways that a signal is more prone to follow, due to the network topology, from a control node to the network end-points, the proportions of the occurrence of *high* and *low* activity for each node in coarse-grained simulations were calculated when the endpoints were highly active. If a node has no correlation with an endpoint, the corresponding proportion is expected to be ∼ 50%. The larger the deviation from this proportion, the larger the involvement of the node within the network.

Any individual node’s activity change (from low to high) influences not only the activity of the endpoints but also that of all other nodes. The average activity of any node *i* as a consequence of an activity change of the control node, *j*, is:

6 $$ \widehat{\Upsilon_{i, \beta(j)=high}}=\overline{SS_{i,\beta(j)=0.1}}, j \ne i  $$

where the bar denotes an average and *SS*_*i*,*β*(*j*)_ the steady-state of node *i* when the control node *j* is set to an independent activity of *β*(*j*). Similarly, $\widehat {\Upsilon _{i, \beta (j)=low}}$ is calculated as:

7 $$ \widehat{\Upsilon_{i, \beta(j)=low}}=\overline{SS_{i,\beta(j)=0.001}}, j \ne i  $$

The ratio $\widehat {\Upsilon _{i, \beta (j)=high}} / \widehat {\Upsilon _{i, \beta (j)=low}}$ represents the effect of the control node’s independent activity change (*β*(*j*)=*low*→*high*) on the steady-state activity of any other node (*SS*_*i*_).

Upon activation of the control node, the statistical association of other nodes that are influenced is represented by the graph’s node area (the larger the area the stronger the association). The colour of the nodes indicates their activity contribution (the darker is a node, the higher is its $\widehat {\Upsilon _{i, \beta (j)=high}} / \widehat {\Upsilon _{i, \beta (j)=low}}$ ratio, and thus the stronger is the signal it can deliver downstream to it).

### Sensitivity analysis in fine-grained simulations

Based on the same mathematical principles as for in the coarse-grained simulations, in fine-grained simulations the majority of the network components were assumed to have a low (resting) activity, while few nodes, identified by coarse-grained simulations as relevant for controlling the network behaviour, were varied over a range of activities (*β*) in small steps (as explained in reference [40] and expanded in Additional file 1: Table S1). This way, a more in-depth, quantitative understanding of the control nodes to the network endpoints is achieved (see Fig. 2). This yielded a more detailed characterisation of those nodes that were critical for controlling the network end-points and consequently relevant for cancer development.

### Principal component analysis and hierarchical clustering

PCA was used as a multivariate analysis to reduce dimensionality of the fine-grained simulations (the *prcomp* function of R was used as a part of the computational method developed by us previously [38, 39]). It was applied to visualise PCA loadings (corresponding to the network components) of steady-state and sensitivity data on a two-component space (as presented in the top panels in Additional file 1: Figures S7–S9). PCA loadings were further classified using hierarchical clustering (the *hclust* function of R was used) and represented in a tree-like structure (dendrogram) whose branches grouped network components according to their similarity over the different simulations (displayed in the bottom dendrograms of Additional file 1: Figures S7–S9).

### Model potential and limitations

A limitation of our approach consists of the fact that quantitative information cannot be obtained for all proteins or complexes of a living model. This prevents precise predictions of the model kinetics and does not allow to take into account time-related properties of the dynamical system such as oscillations [81]. To estimate such quantities is challenging since it can only be determined if a large number of microscopic parameters are available experimentally, while the remaining, unknown parameters are extrapolated by computational methods. This enables to set up mass-action-based models of remarkable predictive power for model systems that were specifically tailored. Examples of this approach could reveal crucial insights for the development of targeted inhibitors [43–48]. Unfortunately, the technical challenges to obtain such high-quality information restrict its applicability to few cellular signalling systems. On the other extreme there are modelling techniques that require only limited, approximate information to make useful predictions based only on node connectivities (e.g., Boolean networks, Petri nets) [49, 50]. In between, our model uses the assumption of steady-state between network components. It only requires minimal information to set up Boolean models, but has the advantage of assuming continuous regulation between nodes, although implemented in a more approximate way compared to detailed mass-action-based models. The advantage of our proposed model is that it enables to study signal transduction pathways for which only sparse information is available, consequently making poorly described diseases networks tractable by simulation. This opens the way for computer-assisted analysis to a majority of complex diseases for which only limited molecular details are available.

Parametric and structural uncertainty were studied in our previous work. The first denotes the changes in the network nodes’ activity as parameters are varied, while the second considers the network qualitative behaviour as a function of the number of nodes considered (e.g., by approximating multiple signalling component as a merged entity). We showed that consistent results were obtained comparing simulations in which parameters were single-valued, to simulations where a numerical ranges was used for each parameter screened. The method demonstrated to be robust against a wide range of parameter variation and therefore proving reliable towards parametric uncertainty [38]. We also showed that we could obtain equivalent results by adding ∼ 50% of nodes and links to a network (note that robustness tests consider highly robust a network able to tolerate variation of 5-20% in the number of the nodes [82, 83]). This proves the method robust with respect to structural uncertainty [39].

Of note, our model can be refined once additional experimental evidence will be made available. Both the elucidation of new signalling pathways interacting with components of our network model (e.g., from omics experiments), as well as the effect of therapeutic inhibitors (and combinations thereof), is information that can be easily integrated to our current model.

## Additional file

Additional file 1 Supplementary Material. Sensitivity profiles and signal flow graphs (Supplementary Figures 1–4). Fine-grained simulations heat maps (Supplementary Figures 5–6). PCA and hierarchical clustering at different levels of PI3K (Supplementary Figures 7–9). Model parameters (Supplementary Table 1). (PDF 3213 kb)

## Abbreviations

ALL: Acute lymphoblastic leukaemia
AKT: Protein kinase B
AML: Acute myeloid leukaemia
BCL2-BAD: BCL2-family protein – BCL2 antagonist of cell death
CDK6: Cyclin dependent kinase 6
CREB: Cyclic adenosine monophosphate-response element binding protein
ELK: ETS domain-containing protein FDA: US food and drug administration
FLT3: FMS-like tyrosine kinase 3
FLT3L: FLT3-ligand
GAB2: GRB2-binding protein
GRB2-SOS: Son of sevenless
HCK: Hematopoietic cell kinase
ITD: Internal tandem duplication
LOA: loss of apoptosis
MEK/ERK: Mitogen-activated ERK kinase / extracellular signal-regulated kinase
mTOR: Mammalian target of rapamycin
NPM-ALK: Nucleophosmin anaplastic lymphoma kinase
PCA: Principal component analysis
PI3K: Phosphoinositide 3 kinase
ODEs: Ordinary differential equations
RSK: 90-kDa ribosomal protein S6 kinase
RTKs: Receptor tyrosine kinases
SHC: SH2-containing sequence proteins
SHIP: SH2-domain-containing inositol phosphatase
SHP2: SH2-domain-containing protein tyrosine phosphatase 2
STAT: Signal transducer and activators of transcription
