# Supplementary material for: Computer simulations of the signalling network in FLT3 +-acute myeloid leukaemia – indications for an optimal dosage of inhibitors against FLT3 and CDK6
Source: BMC Bioinformatics. 2018 Apr 24;19:155. doi: 10.1186/s12859-018-2145-y (PMC5921566; doi:10.1186/s12859-018-2145-y)
Supplement: Supplementary file 1 — Supplementary Material. Sensitivity profiles and signal flow graphs (Supplementary Figures 1–4). Fine-grained simulations heat maps (Supplementary Figures 5–6). PCA and hierarchical clustering at different levels of PI3K (Supplementary Figures 7–9). Model parameters (Supplementary Table 1). (PDF 3213 kb) [file 12859_2018_2145_MOESM1_ESM.pdf]

Combination Therapy for Acute Myeloid Leukaemia – Simulating Optimal Dosage of Inhibitors against FLT3 and CDK6.

Antoine Buetti-Dinh [1,2,3,4,5,*] and Ran Friedman [1,2,*]

[1]Department of Chemistry and Biomedical Sciences, Linnæus University, Kalmar, Sweden antoine.buetti@lnu.se
[2]Linnæus University Centre for Biomaterials Chemistry, Linnæus University, Kalmar, Sweden ran.friedman@lnu.se
[3]Centre for Ecology and Evolution in Microbial Model Systems, Linnaeus University, Sweden
[4]Institute of Computational Science, Faculty of Informatics, Università della Svizzera Italiana, Lugano, Switzerland
[5]Swiss Institute of Bioinformatics, Lausanne, Switzerland
[*]Corresponding author

SUPPLEMENTARY MATERIAL

Additional file 1

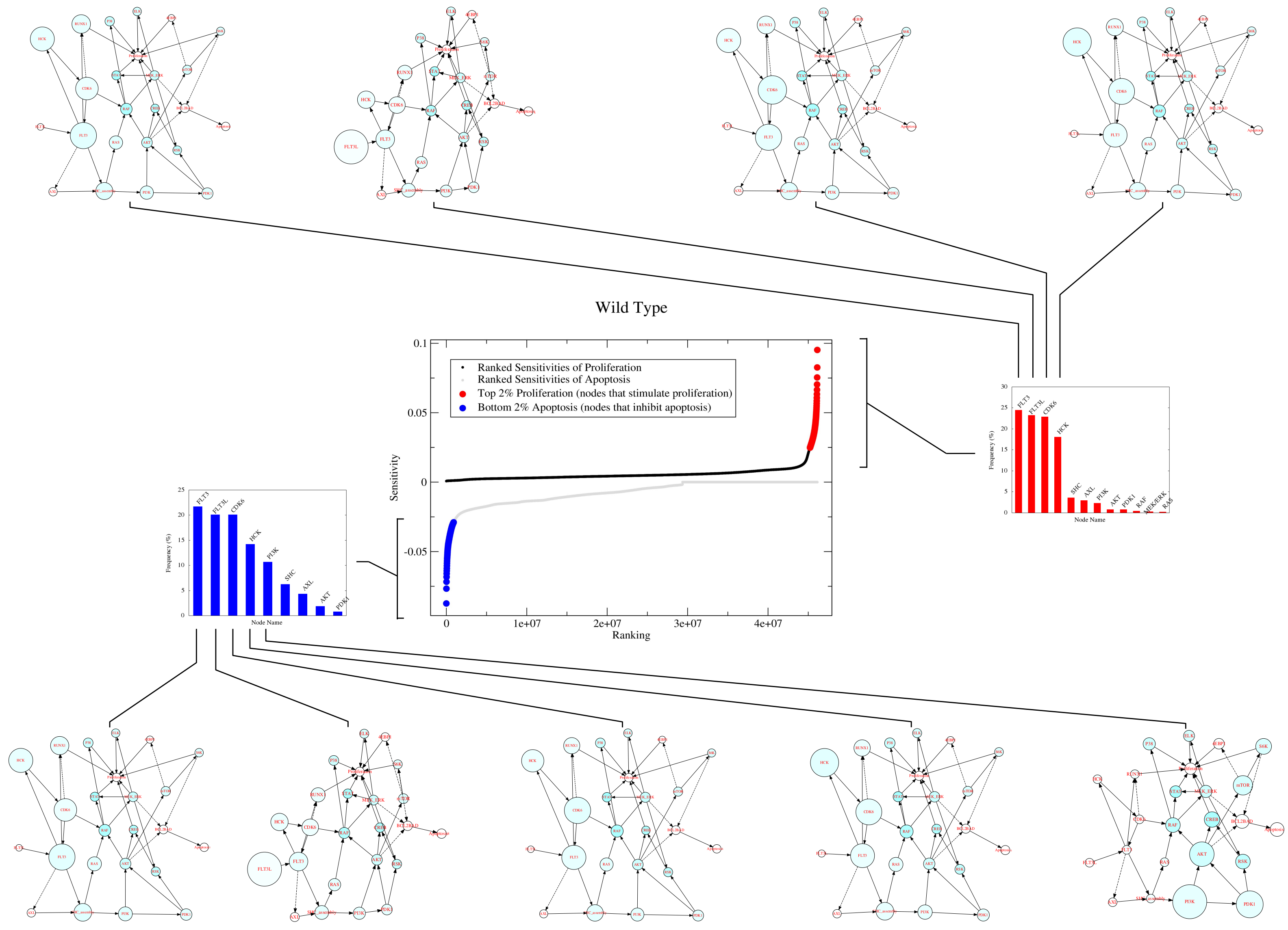

Supplementary Figure 1: **Coarse-grained simulations of the intact network.** Sensitivity profiles shown in the center represent the sensitivity of the network end-points “proliferation” (black) and “apoptosis” (grey) to the activity of the other nodes. A subset of network states induces a strong contribution to the end-points, resulting in significantly increased proliferation (red) or LOA (blue). The network states that contributed most to these subsets were examined to view how often each node is associated to the cancer-promoting state. The results are presented in the barplots. The graphs connected to the bars represent how the signal travels from the control node (indicated on the bar) to the end-points. The size of a node is proportional to its statistical association to the control node, and the colour intensity to the downstream delivered signal flow.

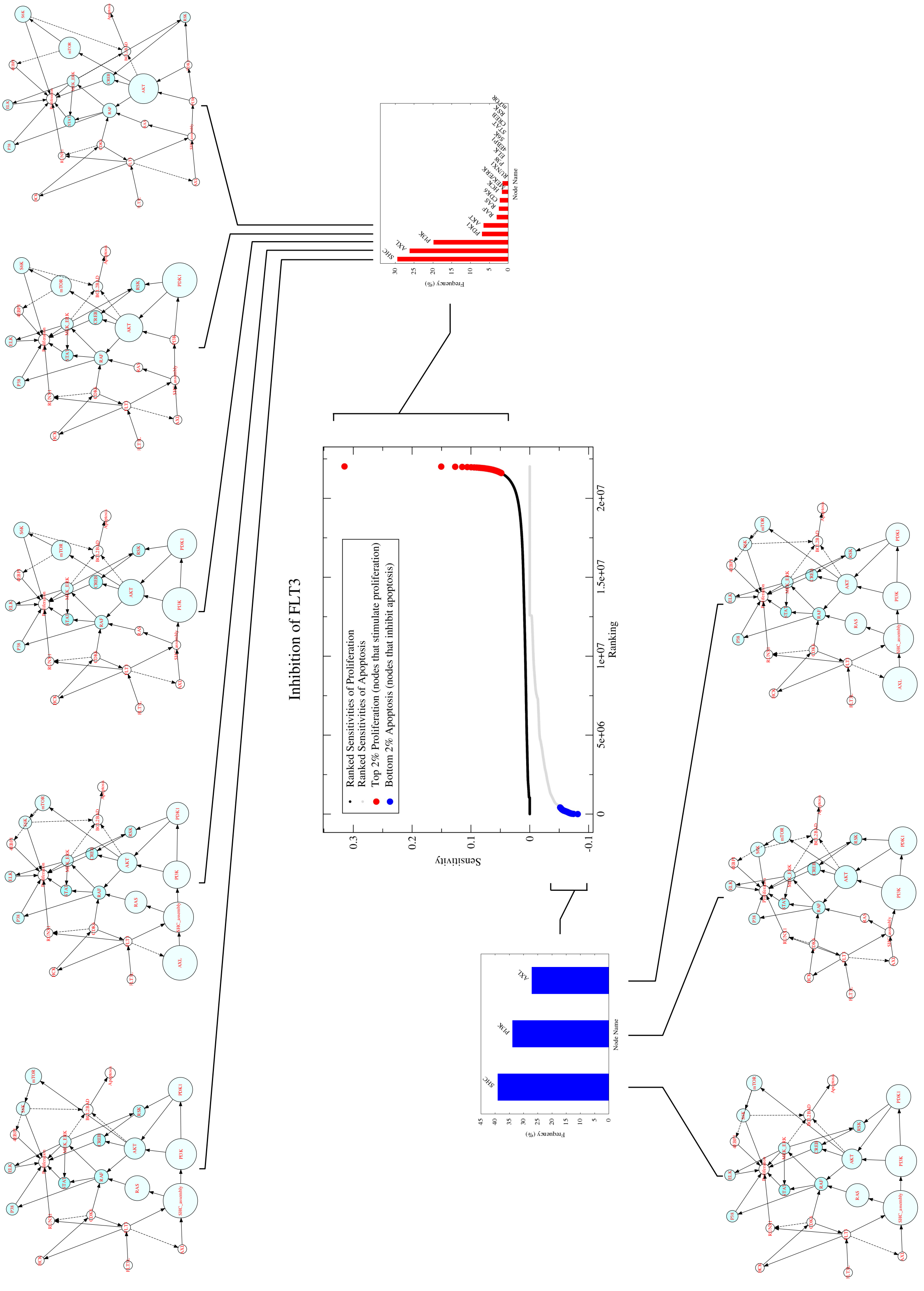

Supplementary Figure 2: **Coarse-grained simulations of the network with inhibited FLT3.** The signal flow graphs, central sensitivity profile plot and flanking barplots are displayed in the same way as in Figure 1

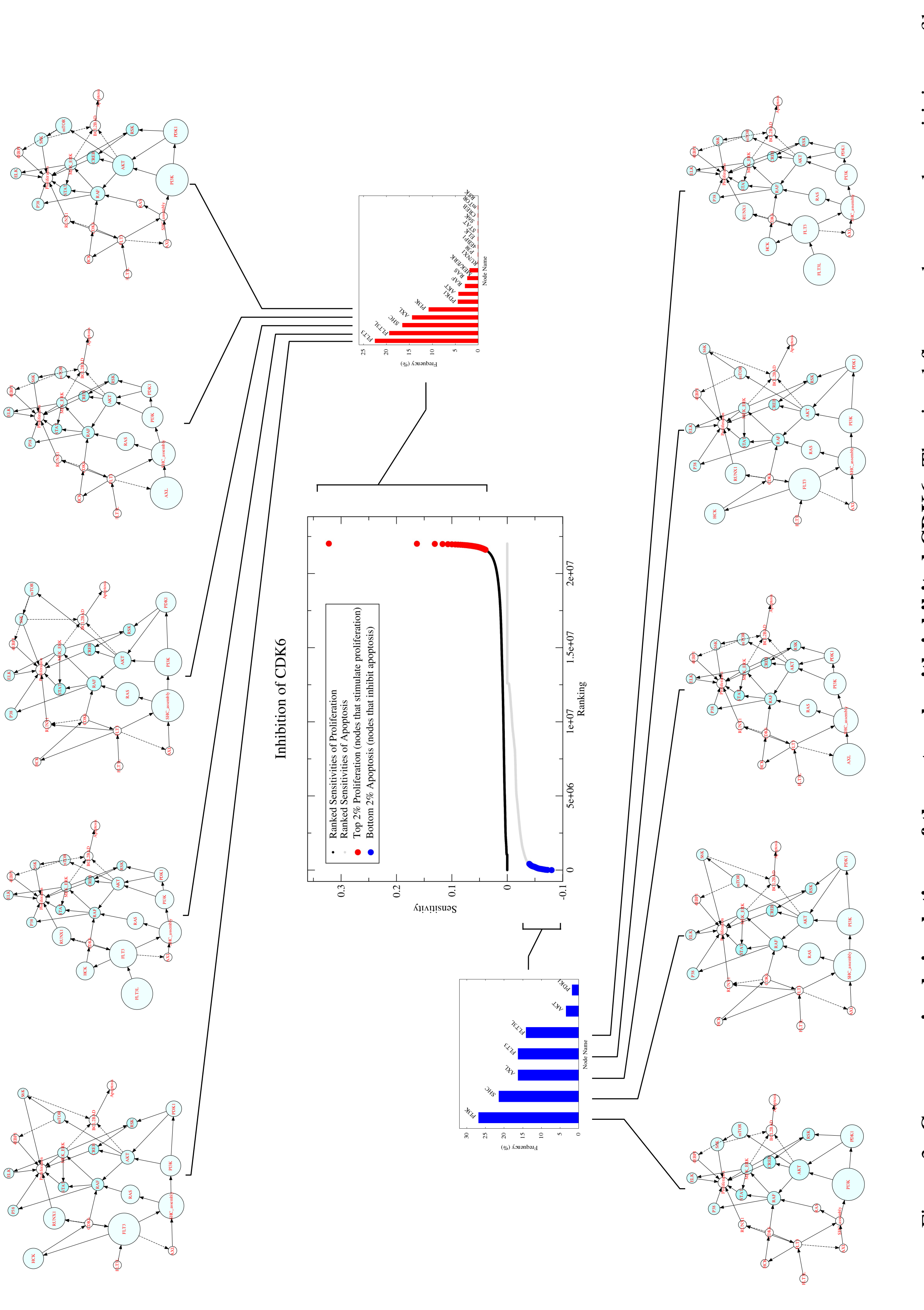

Supplementary Figure 3: **Coarse-grained simulations of the network with inhibited CDK6.** The signal flow graphs, central sensitivity profile plot and flanking barplots are displayed in the same way as in Figure 1

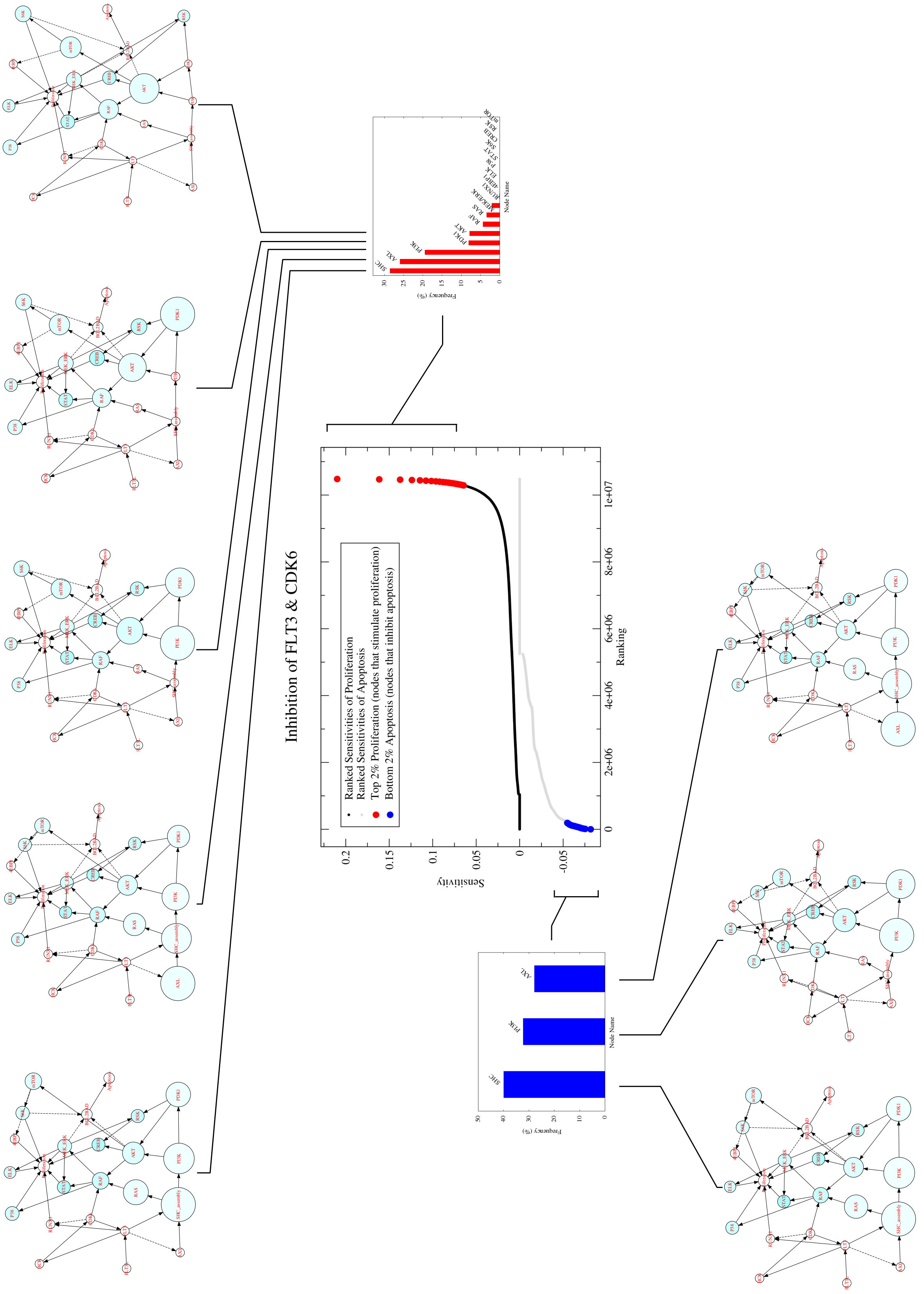

Supplementary Figure 4: **Coarse-grained simulations of the network with inhibited FLT3 and CDK6.** The signal flow graphs, central sensitivity profile plot and flanking barplots are displayed in the same way as in Figure 1

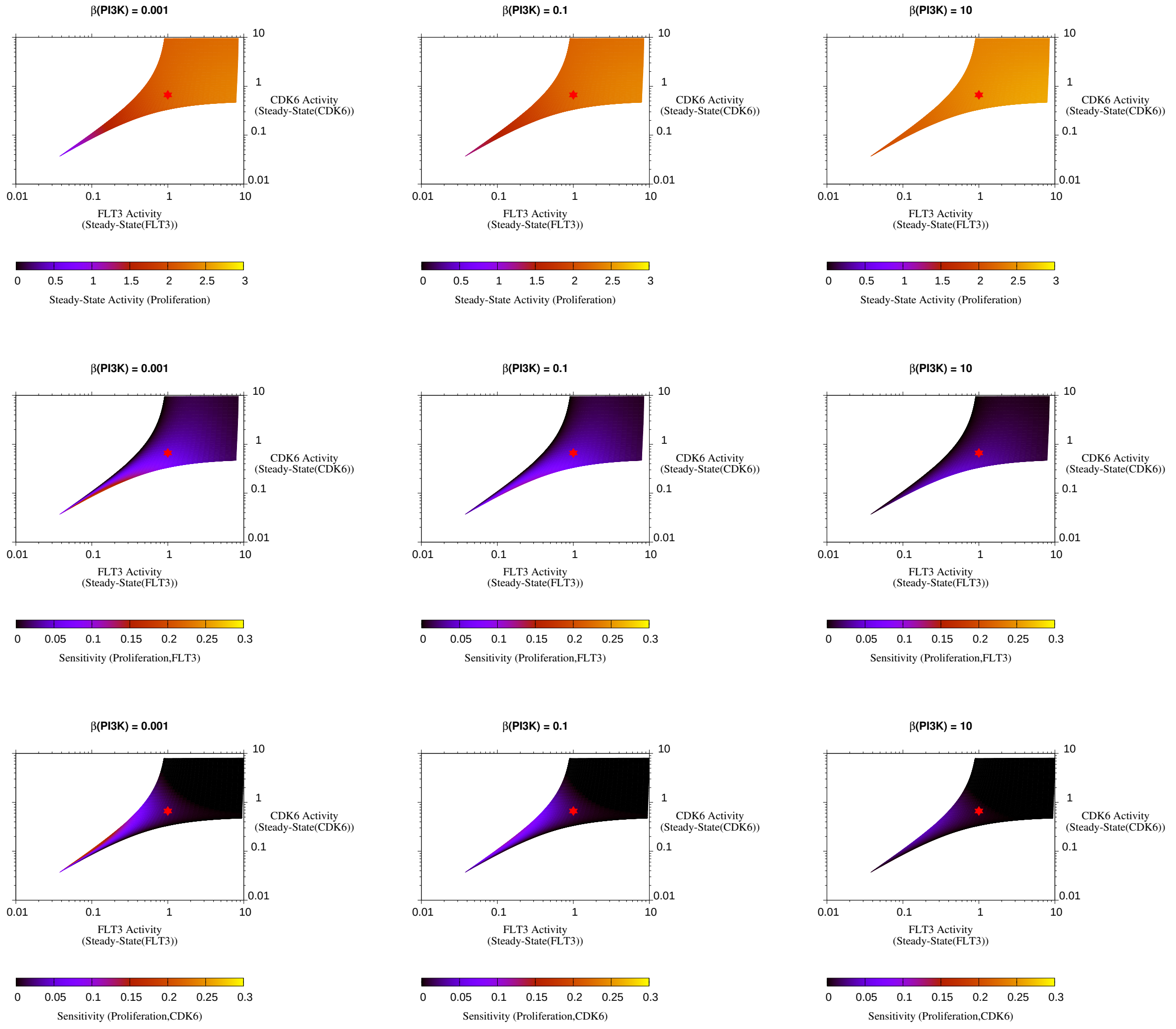

Supplementary Figure 5: **Proliferation heat maps.** 1st row: Steady-state (Proliferation). 2nd row: Sensitivity (Proliferation, FLT3). 3rd row: Sensitivity (Proliferation, CDK6). The red star indicates the point where sufficient inhibition of FLT3 (10-fold inhibition from the maximum) and CDK6 (15-fold inhibition from the maximum) can drive the system to a controllable region of intermediate steady-state levels of both proliferation and apoptosis.

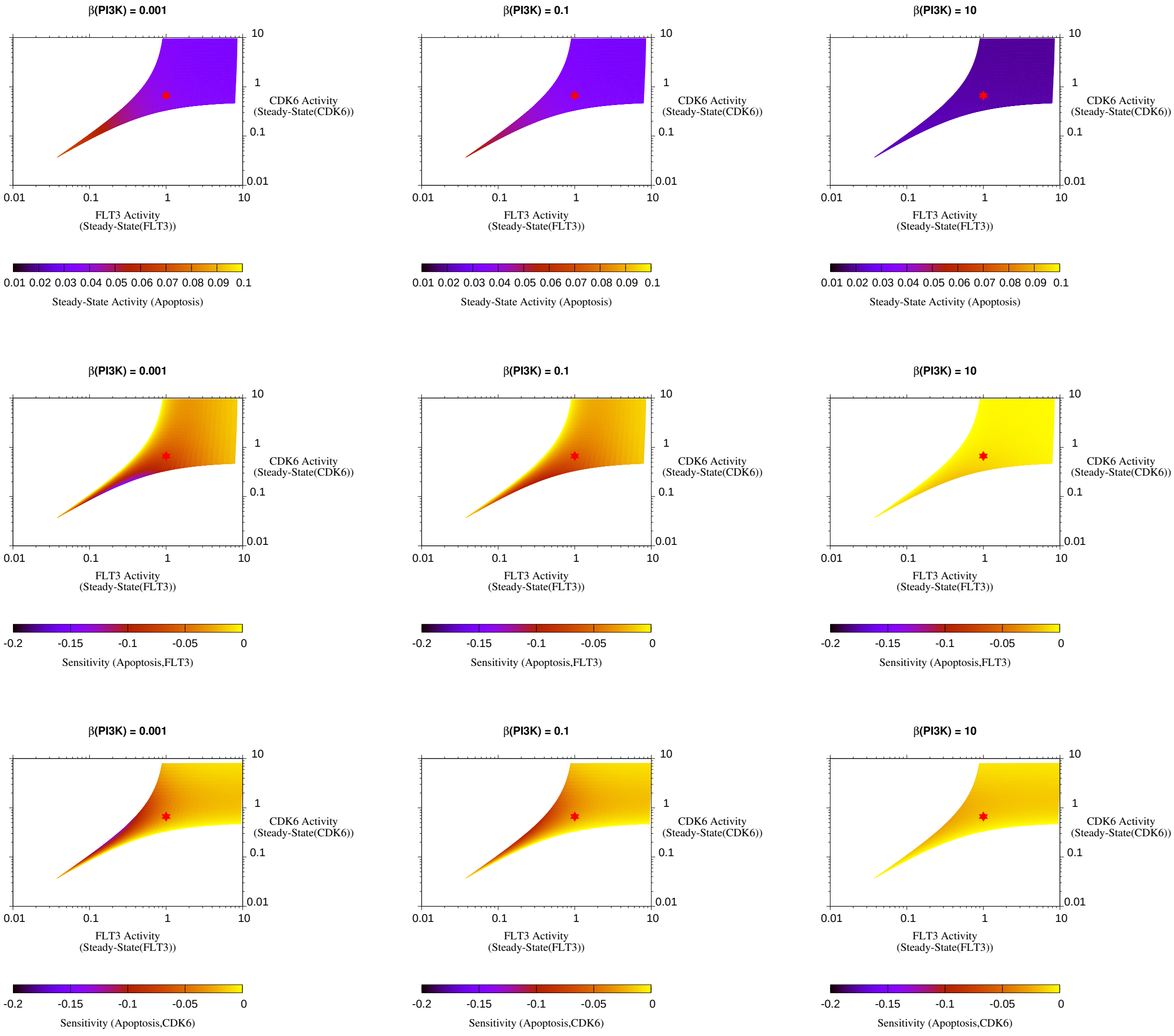

Supplementary Figure 6: **Apoptosis heat maps.** 1st row: Steady-state (Apoptosis). 2nd row: Sensitivity (Apoptosis, FLT3). 3rd row: Sensitivity (Apoptosis, CDK6). The red star indicates the point where sufficient inhibition of FLT3 (10-fold inhibition from the maximum) and CDK6 (15-fold inhibition from the maximum) can drive the system to a controllable region of intermediate steady-state levels of both proliferation and apoptosis.

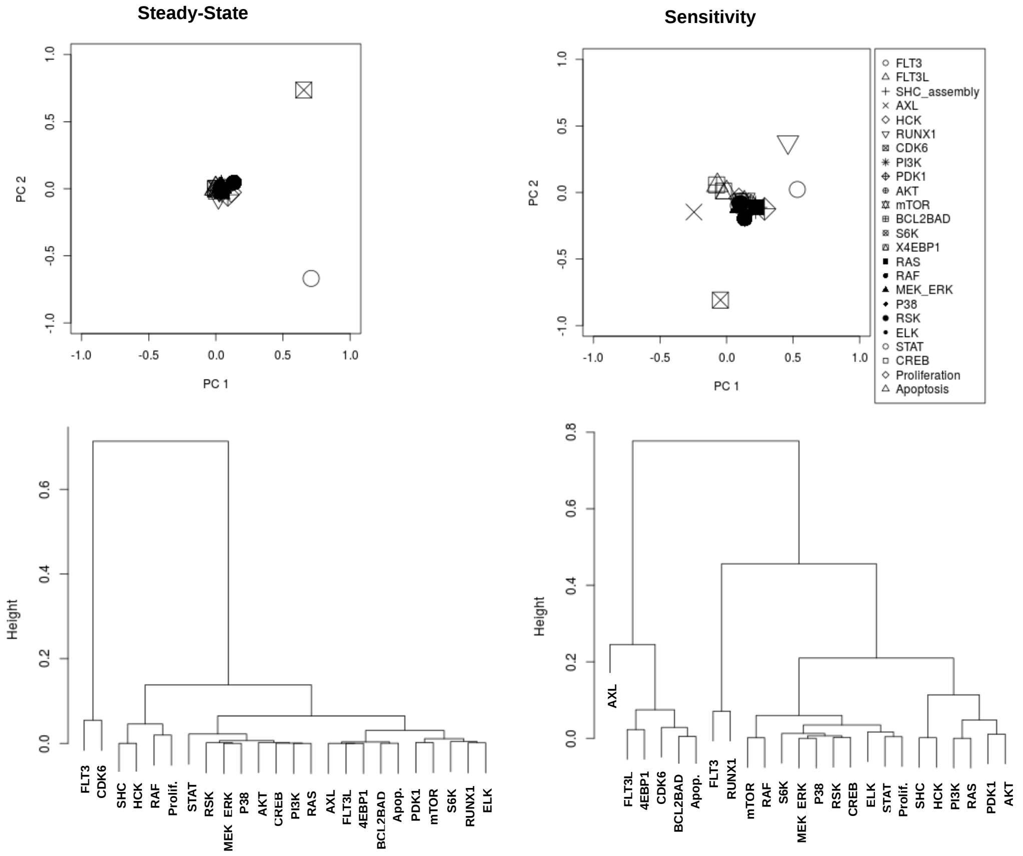

Supplementary Figure 7: **PCA at low levels of PI3K.** Steady-state (left) and sensitivity (right) PCA of the fine-grained simulation dataset at low PI3K ($\beta(PI3K) = 0.001$) corresponding to the red sensitivity surfaces in Figure 2. Hierarchical clustering dendrograms (bottom panels) on the PCA loadings (upper panels).

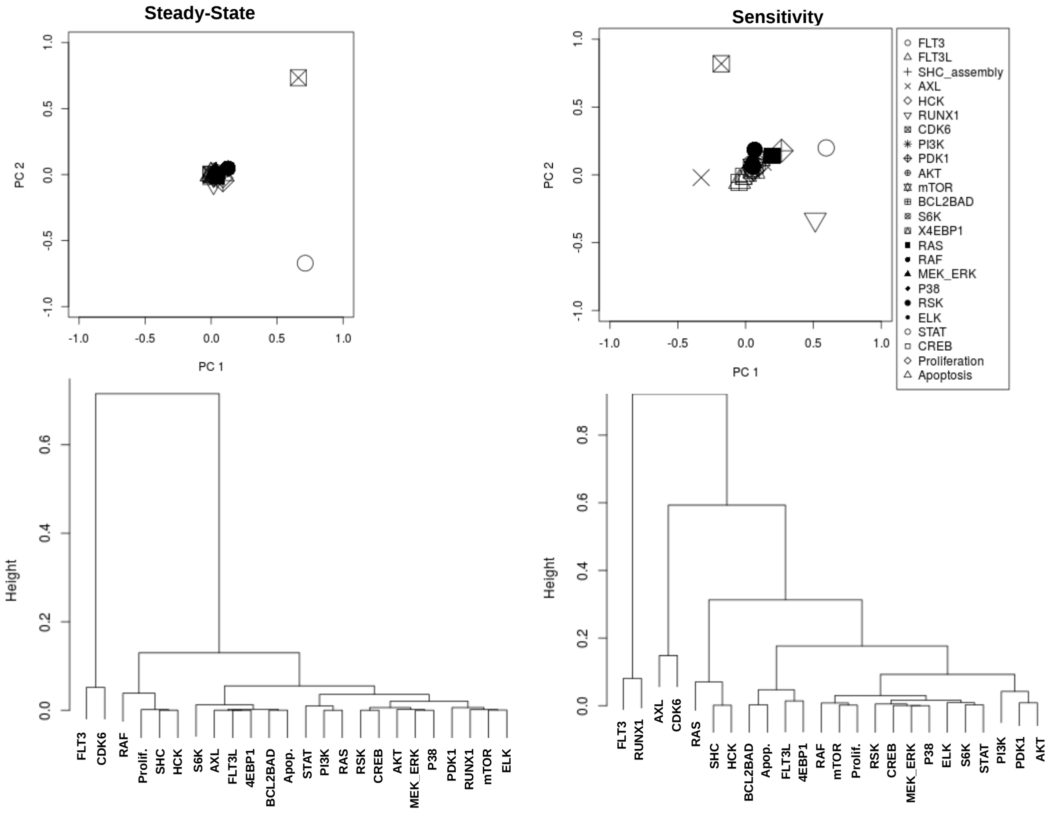

Supplementary Figure 8: **PCA at intermediate levels of PI3K.** Steady-state (left) and sensitivity (right) PCA of the fine-grained simulation dataset at intermediate PI3K ($\beta(PI3K) = 0.1$) corresponding to the green sensitivity surfaces in Figure 2. Hierarchical clustering dendrograms (bottom panels) on the PCA loadings (upper panels).

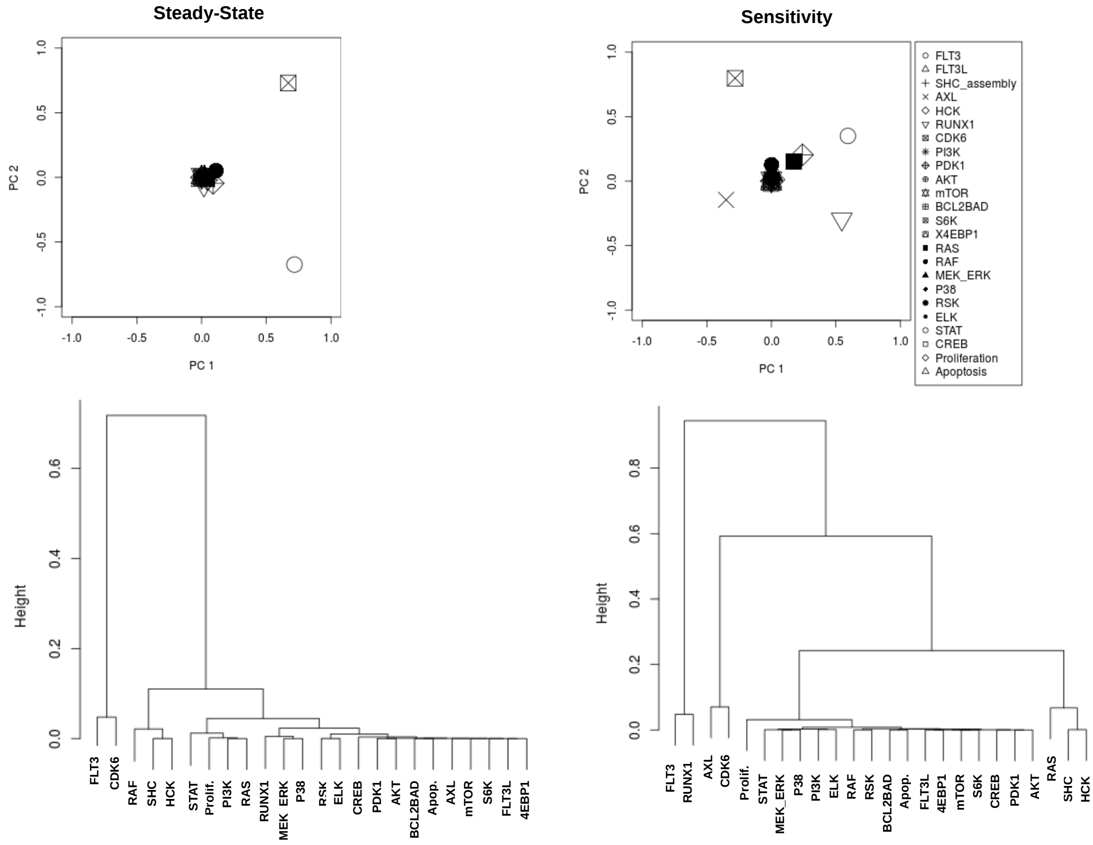

Supplementary Figure 9: **PCA at high levels of PI3K.** Steady-state (left) and sensitivity (right) PCA of the fine-grained simulation dataset at high PI3K ($\beta(PI3K) = 10$) corresponding to the blue sensitivity surfaces in Figure 2. Hierarchical clustering dendrograms (bottom panels) on the PCA loadings (upper panels).

Parameter Name	Coarse-grained Simulations*	Fine-grained Simulations*
$\beta(FLT3)$	10^{-3} - $10^{-1}(100)$	$10^{-3} - 10^{+1}(1.2)$
$\beta(FLT3L)$	10^{-3} - $10^{-1}(100)$	10^{-3}
$\beta(SHC\_assembly)$	10^{-3} - $10^{-1}(100)$	10^{-3}
$\beta(AXL)$	10^{-3} - $10^{-1}(100)$	10^{-3}
$\beta(HCK)$	10^{-3} - $10^{-1}(100)$	10^{-3}
$\beta(RUNX1)$	10^{-3} - $10^{-1}(100)$	10^{-3}
$\beta(CDK6)$	10^{-3} - $10^{-1}(100)$	$10^{-3} - 10^{+1}(1.2)$
$\beta(PI3K)$	10^{-3} - $10^{-1}(100)$	$10^{-3} - 10^{+1}(100)$
$\beta(PDK1)$	10^{-3} - $10^{-1}(100)$	10^{-3}
$\beta(AKT)$	10^{-3} - $10^{-1}(100)$	10^{-3}
$\beta(mTOR)$	10^{-3} - $10^{-1}(100)$	10^{-3}
$\beta(BCL2BAD)$	10^{-3} - $10^{-1}(100)$	10^{-3}
$\beta(S6K)$	10^{-3} - $10^{-1}(100)$	10^{-3}
$\beta(4EBP1)$	10^{-3} - $10^{-1}(100)$	10^{-3}
$\beta(RAS)$	10^{-3} - $10^{-1}(100)$	10^{-3}
$\beta(RAF)$	10^{-3} - $10^{-1}(100)$	10^{-3}
$\beta(MEK\_ERK)$	10^{-3} - $10^{-1}(100)$	10^{-3}
$\beta(P38)$	10^{-3} - $10^{-1}(100)$	10^{-3}
$\beta(RSK)$	10^{-3} - $10^{-1}(100)$	10^{-3}
$\beta(ELK)$	10^{-3} - $10^{-1}(100)$	10^{-3}
$\beta(STAT)$	10^{-3} - $10^{-1}(100)$	10^{-3}
$\beta(CREB)$	10^{-3} - $10^{-1}(100)$	10^{-3}
$\beta(Proliferation)$	10^{-3} - $10^{-3}(1)$	10^{-3}
$\beta(Apoptosis)$	10^{-1} - $10^{-1}(1)$	10^{-1}

* Range of Variation (Fold-Variation Step)

Supplementary Table 1: **Model parametrisation.** Activity units are arbitrary and in the context of signalling proteins roughly translate to the order of 1μM **?** . Values for the network end-points can only be appreciated by comparison.

References

[1] R. Milo, P. Jorgensen, U. Moran, G. Weber, and M. Springer. BioNumbers–the database of key numbers in molecular and cell biology. *Nucleic Acids Res.*, 38(Database issue):D750–753, Jan 2010. [PubMed Central:PMC2808940] [DOI:10.1093/nar/gkp889] [PubMed:19854939].
